# Real-time, neural signal processing for high-density brain-implantable devices

**DOI:** 10.1186/s42234-025-00177-6

**Published:** 2025-07-19

**Authors:** Amir M. Sodagar, Yousef Khazaei, Mahdi Nekoui, MohammadAli Shaeri

**Affiliations:** 1 Integrated Electronic (INTELECT) Research Laboratory, EECS Department, York University, Toronto, ON Canada; 2 Institute of Electrical and Micro Engineering, Center for Neuroprosthetics, EPFL, Geneva, 1202 Switzerland

**Keywords:** Implantable Biomedical Microsystems, Brain Implants, Neural Signals, Signal Processing

## Abstract

Recent advances in the development of intra-cortical neural interfacing devices show the bright horizon of having access to brain-implantable microsystems with extremely high channel counts in the not-so-distant future. With the fabrication of high-density neural interfacing microelectrode arrays, the handling of the neural signals recorded from the brain is becoming the bottleneck in the realization of next generation wireless brain-implantable microsystems with thousands of parallel channels. Even though a spectrum of engineering efforts has been reported for this purpose at both system and circuit levels, it is now apparent that the most effective solution is to resolve this problem at the signal level. Employment of digital signal processing techniques for data reduction or compression has therefore become an inseparable part of the design of a high-density neural recording brain implant. This paper first addresses technical and technological challenges of transferring massive amount of recorded data off high-density neural recording brain implants. It then provides an overview of the ‘on-implant signal processing’ techniques that have been employed to successfully stream neuronal activities off the brain. What distinguishes this class of signal processing from signal processing in general is the critical importance of hardware efficiency in the implementation of such techniques in terms of power consumption, circuit size, and real-time operation. The focus of this review is on spike detection and extraction, temporal and spatial neural signal compression, and spike sorting.

## Brain-implantable microsystems

**Fig. 1:**
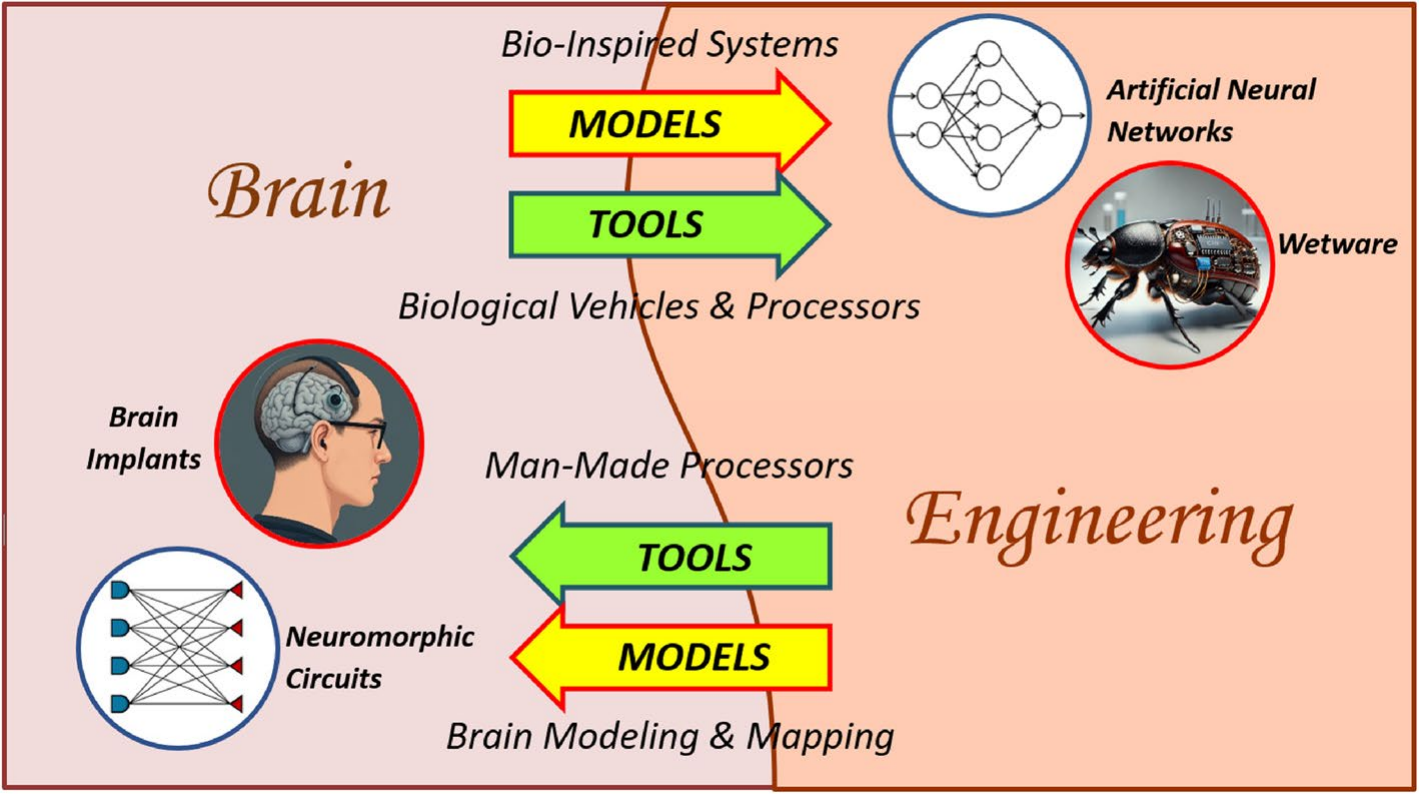
Exchange of models and tools between brain and engineering

Over the past several decades, significant advances have been made in both engineering and brain research. Not only have the progress, findings, and achievements in each field been substantial, but the exchange of ideas and tools between them has also led to the emergence of new interdisciplinary areas with highly valuable and impactful applications. Figure 1 illustrates examples of this fruitful interaction: Models of natural neural networks have inspired researchers on the engineering side to develop innovative methods and novel paradigms for artificially intelligent computing (Poole and Mackworth 2017; S. Russell and P. Norvig 2020; Aggarwal 2018). Additionally, active research lines work towards developing biological processors, made of living neural networks to control man-made systems (Mapelli et al. 2022; Tsur 2021). In return, researchers in neuroscience, medicine, and rehabilitation benefit from a wide spectrum of engineering models and tools to explore the complexities of the brain and cognition, develop effective treatments for previously untreatable diseases, and create prosthetic devices to restore hearing and vision (Counter 2008; Maghami et al. 2014).

**Fig. 2:**
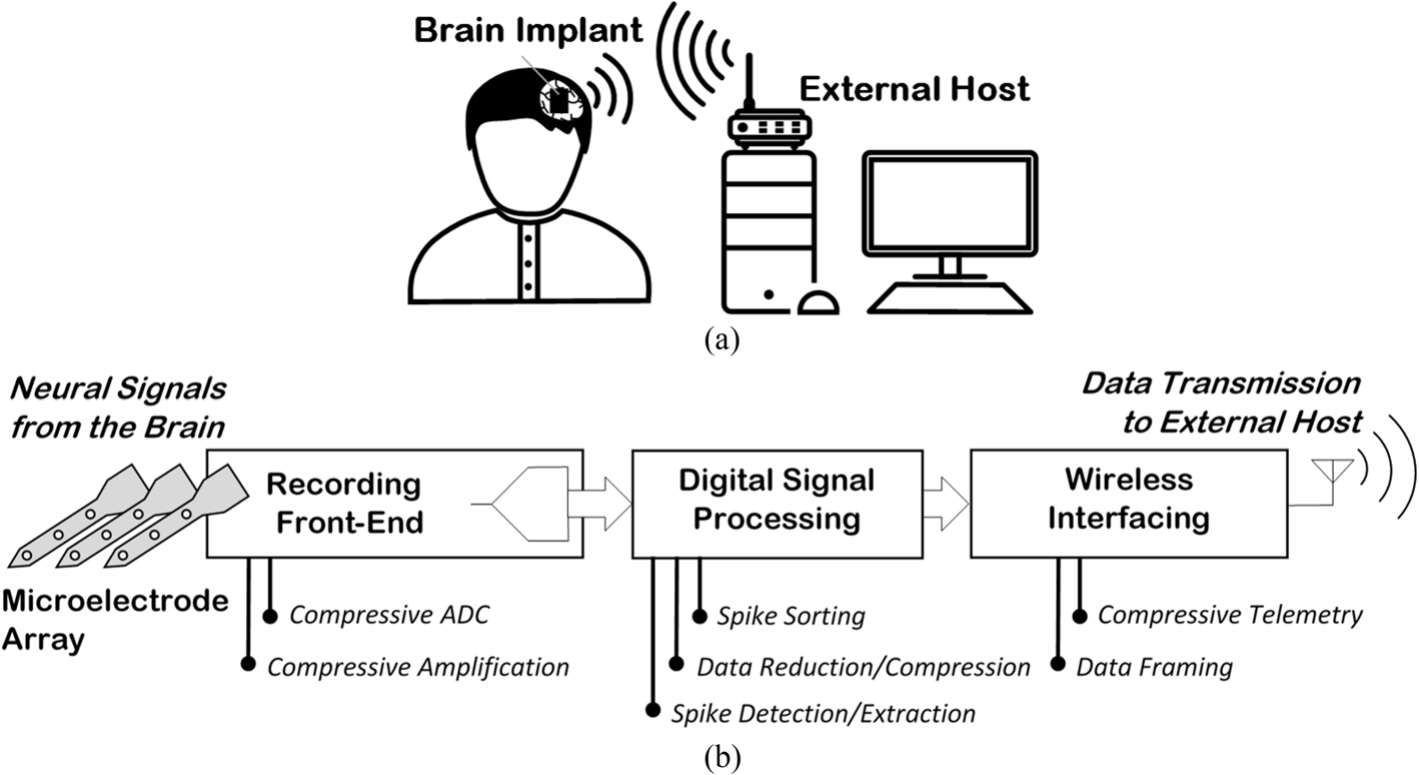
**a** Brain implants for high-resolution interfacing to the brain, (**b**) Simplified block diagram of a neural recording brain implant; Signal processing opportunities along the signal travel path

Brain-implantable microsystems are among the most complicated tools developed to bridge the brain and the outside world. They are stand-alone, miniaturized systems that are developed for interfacing with the biological neural system inside the brain. As a result of residing inside or in close proximity to the brain, such systems provide the possibility of neural interfacing with significantly high temporal and spatial resolutions. As illustrated in Fig. 2a, a brain implant usually communicates with an external host through wireless interfacing. The streaming of neuronal activities from the brain to the outside is referred to as *neural recording*, and triggering or influencing the activities of the brain using the information received from the outside is known as *neuro-stimulation.*

### General overview

Simplified functional diagram of the implantable module of an intra-cortical neural recording system (which is referred to as *brain implant*) is shown in Fig. 2b. The implantable module acquires neural signals using a microelectrode array, and then pre-amplifies and filters them in the analog domain. The recorded signals are subsequently digitized to take advantage of digital signal processing procedures as required, also benefit from digital communication techniques for the telemetry of the recorded data to the external module. The microelectrode array, the analog signal preconditioning circuits, and the analog-to-digital converter altogether are usually referred to as the *recording front-end* of the system.

### Signals of interest

In the brain neuronal network, neurons encode information by modulating the pattern of spiking activities (a.k.a., action potentials’ or simply ‘spikes’). All-or-none spiking activities, resulting from rapid changes in the cell membrane voltage are the essential components in information transmission in the central nervous system through electrochemical signaling (Kandel, et al. 2012). Intra-cortical spiking activities can be monitored with high temporal and spatial resolution by placing an electrode array in the vicinity of neurons (Steinmetz et al. 2021). Such activities are mainly used for prosthetic and rehabilitation applications.

**Fig. 3:**
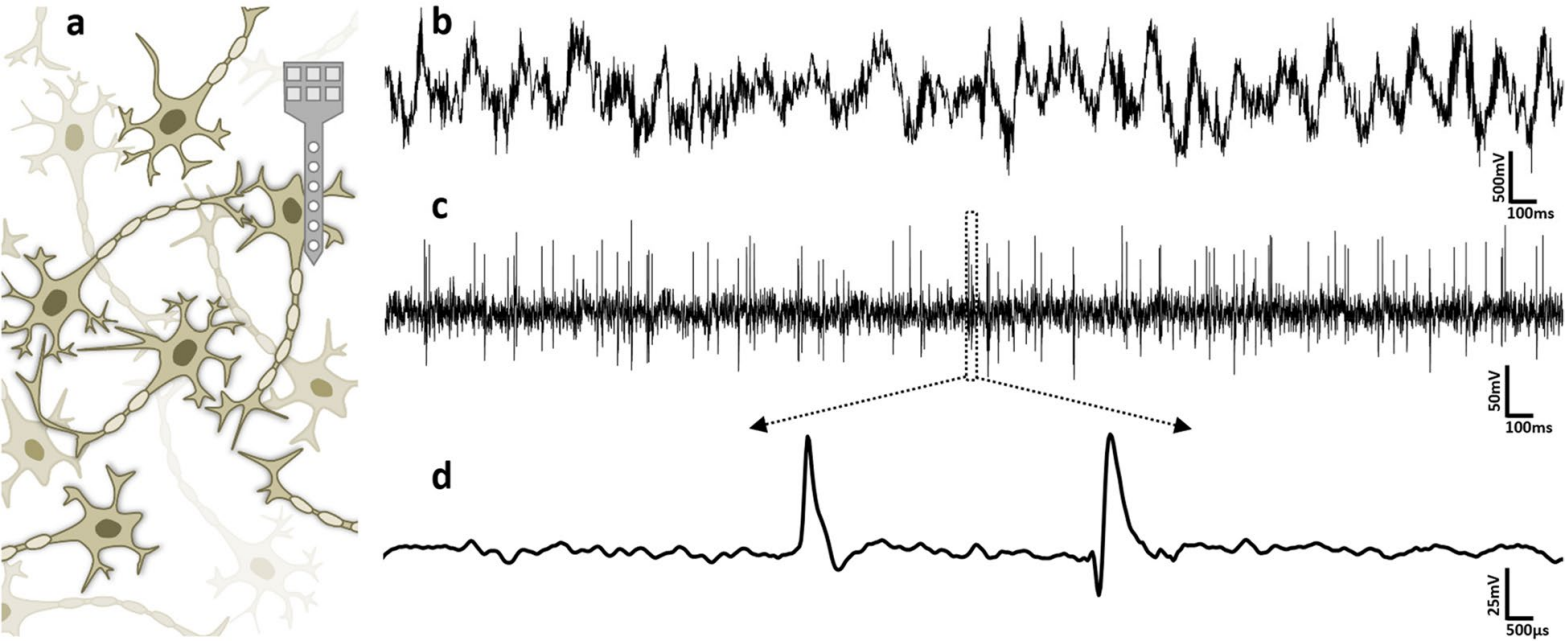
Extra-cellular recording of neuronal activities: (**a**) Acquiring neural signals from a population of neurons using microelectrode arrays; (**b**) the recorded signal; (**c**) the signal after removing the local field potential. This is a mixture of neural spikes and background noise; and (**d**) close-up view of a window on the signal shown in (**c**) containing two action potentials (spikes)

As shown in Fig. 3, an intra-cortically recorded neural signal comprises three major components: ‘action potentials’, ‘local field potentials (LFPs)’, and ‘background noise’. *Action potential* is an electrochemical impulse that is generated by a neuron as a result of processing the impulses it receives from other neurons. Electrically recorded from the vicinity of the firing neuron, an extra-cellular action potential is an amplitude fluctuation within the range of tens to hundreds of micro-Volts that occurs over the course of typically 1 ms~2 ms. The LFP is a low-frequency signal component that is believed to be the average of neuronal activities occurring far away. Even though in some cases researchers acquire the information of interest from the LFP, action potentials are predominantly a key source of information in intra-cortical neural signals. A neural signal is also superposed with the background noise, which degrades the quality of the signal and consequently lowers the performance of signal processing.

To preserve the high spatial resolution of action potentials in intra-cortical recording and at the same time remove LFPs and other non-useful low-frequency components (*e.g.*, DC offset and also offset drift of signal preconditioning circuits), neural signals are pre-amplified and filtered with a low cut-off frequency of 100–300 Hz and a bandwidth of 6 kHz-10 kHz. To be prepared for handling and processing in the digital domain, such signals are sampled at a rate of up to 20–30 kSample/sec. and quantized with a typical resolution of 8–10 bits (Zhang et al. 2023).

### Functions and applications

The first step in studying brain activities is to record brain signals using *neural interfaces* (Razmpour, et al. 2016). Since information is distributed across the brain network, neuroscientists require high-density recording of neural signals. They process the recorded signals in order to extract informative content and eliminate task-irrelevant information (Shaeri and Sodagar 2023). In addition, on-chip processing reduces the volume of the recorded data, enabling efficient data transfer with low transmission power. Next-generation neural interfaces will be designed for prosthetic and therapeutic applications. Prosthetic neural interfaces need to be portable hardware-efficient devices that can restore lost brain functions (*e.g.*, sensation, movement, and communication abilities) (Shaeri et al. 2024; An et al. 2022; Maghami et al. 2016). Being capable of both neural recording and stimulation, closed-loop neural interfaces detect neuro-markers of brain disorders and stimulate the brain accordingly to either suppress abnormal brain activity (*e.g.*, seizure or tremor) or to aid in recovery (*e.g.*, stroke).

## Towards high-density neural recording implants

**Fig. 4:**
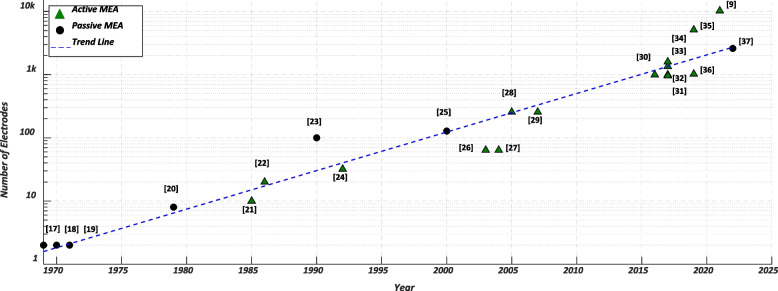
Exponential growth of the number of electrodes on neural recording arrays over the past 50 + years

**Fig. 5:**
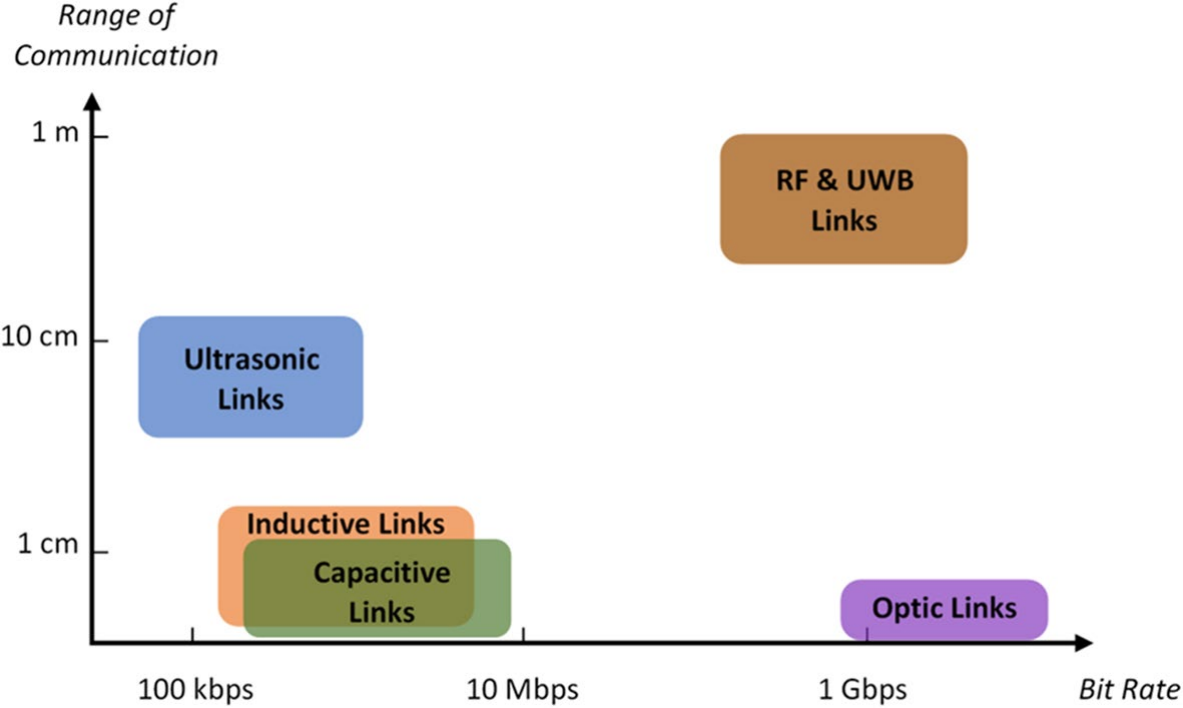
Bit rate and range of communication for different links

Implantable microelectrode arrays have played a key role in the advancement of neuroscience and brain-computer interfaces (BCIs) by enabling high-density and high-resolution recording, which is essential for a comprehensive understanding of and effective interfacing with the brain (Patrick-Krueger et al. 2024). Figure 4 shows the evolution of microfabricated neural recording microelectrode arrays in terms of the number of recording sites (a.k.a. *electrodes*) over the past 50 + years. From the introduction of the first microfabricated silicon probes using microelectronic technology in 1969 (Wise et al. 1969, 1970; Wise and Angell 1971) to the mid-2000 s, the main engineering focus was on realizing complete systems capable of fully wireless neural recording (May et al. 1979; Najafi et al. 1985; Najafi and Wise 1986; Norman 1990; Ji and Wise 1992; Hofmann et al. 2000; Csicsvari et al. 2003; Olsson et al. 2004; Olsson and Wise 2005; Aziz et al. 2007). With recent reports on the fabrication of microarrays of around 1000 ~ 10000 + electrodes (Steinmetz et al. 2021; Lopez et al. 2016, 2017; Jun et al. 2017; Herbawi et al. 2017; Raducanu et al. 2017; Wang et al. 2019; Angotzi et al. 2019; Saleh et al. 2022), the bottleneck in the realization of high-density brain implants has now shifted to ‘real-time handling’ and ‘wireless transmission’ of the data they record. The former is a necessity on the application side, and the latter is a technical requirement stemming from both the limited bandwidth allocated for wireless interfacing (Radio spectrum allocation n.d.) and the restrictions in the electric power budget that is available to a brain-implantable device. The typical bit rate and communication range for different types of wireless data telemetry links—ultrasonic, radio frequency (RF), ultra-wideband (UWB), inductive, capacitive, and optical—used in brain implants are shown in Fig. 5.

### Inevitable need for on-implant signal processing

The most effective solution that can confront the aforementioned “*recording density-transmission bandwidth*” dilemma is the employment of proper signal processing techniques on the implant prior to transmitting the recorded data to the external world. Signal processing in this context mainly aims at reducing the volume of the data being transmitted off the neural recording implant with no or minimal loss of recorded information. Represented by a separate task in the implant functional block diagram shown in Fig. 2b, digital signal processing refers to a wide spectrum of signal-level techniques that will be discussed in this paper.

### On-Implant signal processing requirements

In most applications (*e.g.*, neuroscience research, brain mapping, and brain-machine interfacing), recording a sufficiently high volume of data is essential for collecting conclusive information from the brain. This is the key drive behind extensive research towards realizing high-density neural recording brain implants. Given the strict restrictions in the design and fabrication of brain-implantable devices, recording huge amounts of data and wirelessly transmitting them off the implant is not a trivial engineering task and faces significant implementation challenges.

What distinguishes on-implant neural signal processing from general ‘signal processing’ is the challenges associated with their application-specific requirements as well as their integration into a physical device. The key technical requirements for signal processing on high-density neural recording brain implants can be categorized as follows:

#### Signal processing

Although the neuronal activities acquired from the brain contain some degree of redundancy (*i.e.,* irrelevance to the task at hand) in the information they carry, they also hold crucial information that needs to be retained. This makes the choice of processing techniques critical for preserving useful information while disregarding the redundancies. The concept representing this aspect of the processing of neural signals on brain implants is the accuracy of computation. From a computational perspective, on-implant signal processing should be computationally effective and of low complexity, especially when it comes to handling a huge number of concurrent signals in parallel (or in a quasi-parallel way). Moreover, robustness of processing tasks against the inevitable variabilities that exist in the realm of neural signal processing is a necessity. That includes (but not limited to) the variability of spike waveshapes (from one neuron to another, from one subject to another, and from one recording trial to another), and the background noise content of the signal being processed.

#### Circuit design

The circuit-level implementation of on-implant signal processing techniques needs to comply with a variety of hardware budget restrictions. The hardware implementation of on-implant neural signal processing techniques will, therefore, need to be of extremely low complexity (Marblestone et al. 2013). Moreover, take into consideration the fact that an implantable device is energized either using an embedded battery or through wireless power transfer. Either way, the electric power available to an implant is limited, leaving all internal circuitry (including on-implant signal processors) with a serious restriction in the allocated power budget. Regardless of physical modality, exposure of living tissue to a large amount of electromagnetic or ultrasonic energy for wireless power telemetry arises tissue safety concerns. This concern turns into a challenge when noting that the small size of implants requires the transfer of energy with significantly high density. Tissue safety standards enforce power density limits to ensure that power telemetry does not cause damage to living tissues ("IEEE Std C95.1–2005 2006; FDA 2008). Moreover, part of the electrical energy used by any circuit or system is converted to thermal energy, and implantable devices are no exception. This causes some extent of temperature rise, which may harm surrounding tissues (ISO 14708-1:^2014^). The importance of this recommendation is more highlighted when noting that the hardware proposed for the realization of a given processing task is expected to be expanded to handle up to several thousands of usually concurrent signals.

#### Speed

According to functional requirements in almost all application areas (especially for real-time prosthetic applications) on one hand, and the impracticality of envisioning a huge memory capacity for locally storing the recorded data on the other hand, brain-implantable devices must stream neural signals in real time. As a result, ‘on-the-fly’ operation is usually a necessity for on-implant signal processing. What makes the matter more challenging are the restrictions implants face in the speed of data transmission to the outside world, mostly stemming from the limited bandwidth of wireless communication.

#### Physical implementation

In general, an implant is required to be of small physical size. This is simply because no free space is envisioned inside the body for such a foreign object. Moreover, depending on where it is supposed to be implanted, (a) the device should be of a proper shape and form factor, and (b) the details of their assembly and integration are also determined accordingly. As a general guideline, all internal circuitry (including on-implant signal processors) should be of the least possible complexity and size, and with preferably no off-chip parts.

### On-implant signal processing categories

As mentioned previously, neural spikes are believed to convey the key information in neuronal communication. Therefore, preservation of the integrity of neural signals in terms of the main attributes of neural spikes (*e.g*., their occurrence, waveshape or class, and timing) is a hardline in the recording and processing of neural signals on a brain implant. The neural signal processing techniques that are used on brain-implantable devices can mainly be categorized under three general types: *spike detection and extraction*, *data compression*, and *spike sorting*. An overview of the techniques employed for implant-appropriate implementation of these processing tasks is presented in the following sections.

The neural signal processing techniques used in brain interfaces can primarily be classified into three general categories: *spike detection and extraction*, *data compression*, and *spike sorting*. The following sections present an overview of the techniques employed for the implant-appropriate implementation of these processing tasks.

## Spike detection and extraction

The typical spiking firing rate in an intra-cortical neural signal ranges from 10 to around 150 spikes/s. Given that the time course of a typical spike is 1 ~ 2 ms, a neural signal is, therefore, a sparse signal in which only a small fraction of the recorded data carries useful information (*i.e.*, spikes). In applications such as prosthetic devices, only detecting the occurrence of spikes suffices to decode the information of interest (Nason et al. 2020).

Neural spike detection is usually performed through hard thresholding, in which a certain attribute of the signal is compared with a given threshold level. The attributes used for this purpose are the amplitude of the signal in the time domain (Barati and Sodagar 2010), its absolute value (Obeid and Wolf 2004), the nonlinear energy operator (NEO) of the signal over a narrow window in time (Yang and Mason 2014), the magnitude of low-pass (approximate) coefficients of the signal in the discrete Haar wavelet space (Shaeri et al. 2011), and a measure of the smoothness of amplitude variations (Mirzaei et al. 2020). Moreover, the dual spike detector utilized both the original and smoothed signals, followed by NEO transformation, to accurately distinguish spiking activities in both high- and low-noise cases (Guo et al. 2022). The threshold levels used for spike detection can be defined either manually by the user (Sodagar et al. 2007), or automatically by the recording system according to the background noise content of the signal being recorded (Turcotte and Gosselin 2015). In the latter approach, an adaptive threshold level is set about 3 ~ 7 times the standard deviation of the background noise above (or below) the baseline level of the signal (Razmpour et al. 2016; Barati and Sodagar 2010). Adaptive threshold generation and spike detection have been implemented in both analog (Barati and Sodagar 2010) and digital domains (Olsson and Wise 2005; Gagnon-Turcotte and Gosselin 2015; Zhang et al. 2023). Moreover, (Yang and Töreyin 2024) introduces a current-mode analog signal processing circuit with a spike enhancement filter designed to improve the SNR.

**Fig. 6:**
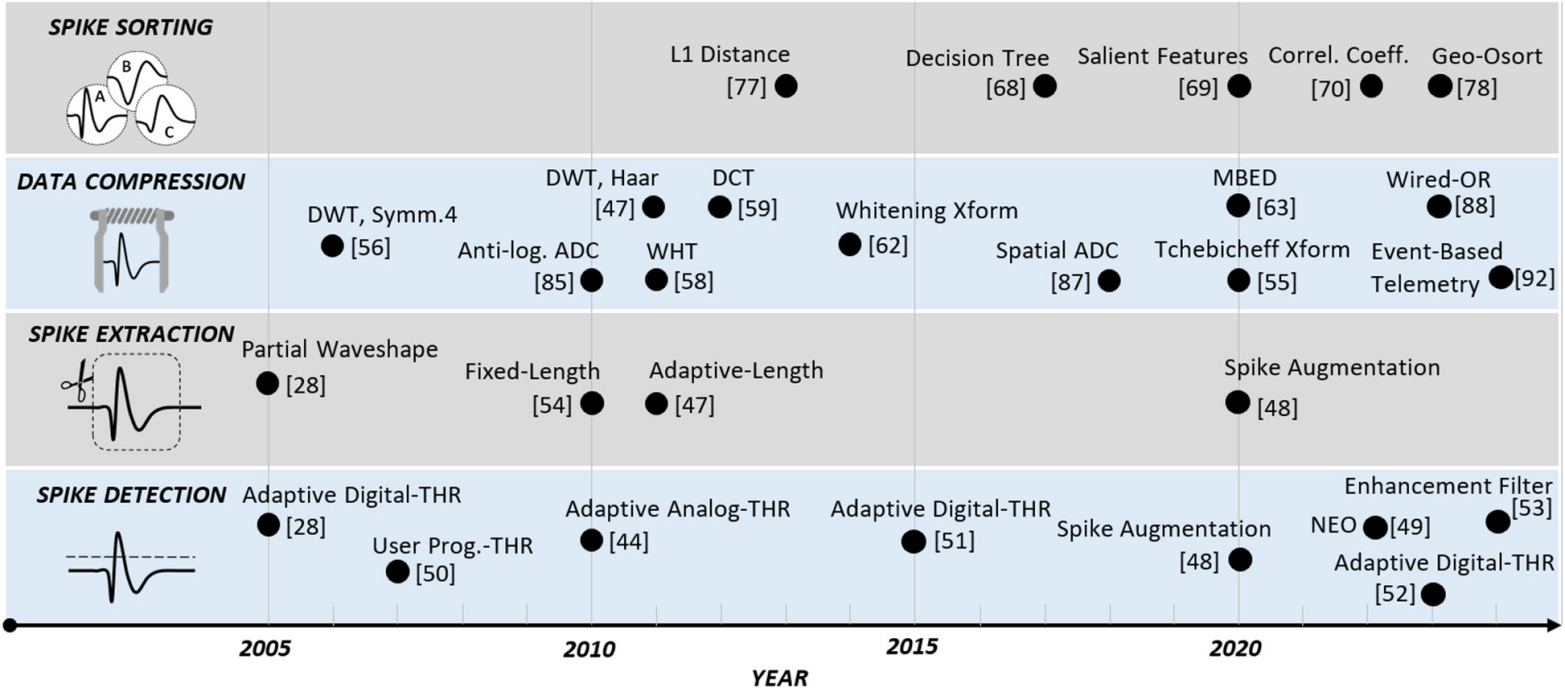
Chronological summary of on-implant neural signal processing techniques

In applications where spike wave shapes are required, neural spikes are extracted from the recorded signal. After its occurrence is detected, a spike is extracted in different ways: In (Olsson and Wise 2005), only the parts of the spike that go beyond a pair of adaptive threshold levels (symmetrically defined around the signal baseline or mean value) are extracted. In this approach, what remains from the spike waveshape hardly resembles a typical spike waveshape. To preserve the integrity of spikes, it is suggested in (Gosselin and Sawan 2008) that once a spike is detected, a sufficiently wide segment of the signal that accommodates the spike is extracted and telemetered off the implant. The length of this segment is fixed in (Gosselin and Sawan 2008), while is adaptively determined according to the length of the spike in (Shaeri et al. 2011; Shaeri and Sodagar 2015). Figure 6 presents a chronological summary of the pioneering works reported over the past two decades not only in spike detection and spike extraction, but also in data compression and spike sorting (to be explained in the next sections).

## Data compression

The term *data compression* (or *signal compression*) is more appropriate when an entire neural signal (comprising neural spikes, inter-spike background noise, and sometimes LFPs) is compressed. When a signal compression technique is applied on extracted spikes, the term *spike compression* is usually used.

To reduce the extent of recorded data while preserving the key information in neural signals^1^, there are two general categories of approaches: (a) *data reduction* techniques, in which part of the data conveying no or insignificant information is discarded, and (b) *data compression* techniques, which suggest more compact representation for the recorded data. However, it should be added that in neural signal processing, the terms ‘data compression’ and ‘data reduction’ are interchangeably used. Hereafter, the term ‘data compression’ is used in this context, applied to the techniques used for both purposes.

**Fig. 7:**
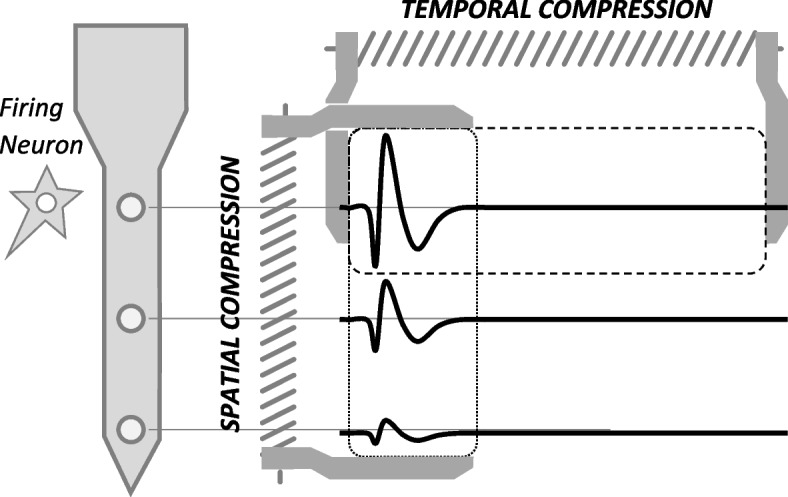
Compression of neural signals, temporal vs. spatial

From a signal-level viewpoint, compression of neural signals can be either temporal or spatial. As illustrated in Fig. 7, *temporal compression* is the act of compressing a time series of signal samples recorded on a given channel. On the contrary, *spatial compression* is applied on the samples (or windows of samples) recorded on multiple channels concurrently at a given instant of time (or over a given time window).

### Temporal compression

Temporal compression is usually performed by finding a more efficient representation for the signal than its original representation. Perhaps the simplest mathematical technique to temporally compress a neural signal is to start from an initial value at the background level of the signal and report the incremental variations of the signal rather than its instantaneous values. The so-called ‘delta approach’ in Aziz et al. (2007) is applied on the entire neural signal and achieves lossless data reduction by reporting only the amplitude difference between every consecutive samples rather than their actual amplitude values.

Higher data reduction efficiency is achieved by first discarding inter-spike background noise and then applying compression techniques on extracted spikes (previously introduced as ‘spike compression’). Given that spikes are sparse events in intra-cortical neural signals, discarding inter-spike intervals helps achieve significant data reduction. As extensively discussed in Shaeri and Sodagar (2023), mathematical transforms help in different ways achieve better performance in signal compression. They may enhance the discrimination between neural spikes and the background noise, extract the key information conveyed by neural signals, or map the signal to a different space where the information of interest is represented in a more compact way. In most cases, all a transform does is redistributing the information or energy of the signal in such a way that the majority of the signal energy (*e.g.,* the energy of spikes in a neural signal) is aggregated in a limited number of coefficients and other coefficients convey an insignificant portion of the signal energy. As a result, one can achieve considerable data reduction in the ‘transform domain’ (as opposed to the time domain) through discarding the coefficients that are of insignificant energy (Farsiani and Sodagar 2020).

On-implant, temporal, transform-based compression of neural spikes goes back to 2006, when Oweiss, et al., suggested a digital neural signal compressor based on the discrete wavelet transform (DWT) with symlet4 basis function (Oweiss, et al. 2007). The existence of multiple digital multipliers in the digital implementation of this transform leads to large chip area and high-power consumption, neither of which is favorable for on-implant signal processing. To realize hardware-efficient transform-based spike compression yet achieve comparable signal processing performance, the discrete Haar wavelet transform (DHWT) (Shaeri and Sodagar 2015), the Walsh-Hadamard transform (WHT) (Hosseini-Nejad, et al. 2011), and the discrete cosine transform (DCT) (Hosseini-Nejad, et al. 2012) are subsequently proposed, all implementable with no need for digital multipliers. To achieve highly efficient data compression, a truncated approximate Tchebycheff transform has recently been proposed in (Farsiani and Sodagar 2020), which is optimized for minimal energy leakage between transform coefficients and is implemented using additions, subtractions, and scaling by small integers (± 1, ± 2, and ± 3). It should be added that in transform-based compression, even if the mathematical transform itself is not compressive, it can provide the possibility of data reduction through redistributing the signal energy. In this case, techniques such as truncation and thresholding in the transform domain result in significant data reduction. As a different approach, the work in (Nekoui and Sodagar 2022) first breaks the neural spike into multiple segments, and then uses a polynomial approach to modeling the curvature of each spike segment. What is telemetered off the brain implant is the timing and amplitude of the start and end samples of spike segments (referred to as ‘*salient samples*’) rather than all samples of the segments. On the external side of the recording system, spike segments are first retrieved by regression, and then the spike waveshape is reconstructed by concatenating the retrieved spike segments. This technique not only effectively reduces the volume of the data representing the spike under study, but also helps significantly denoise the spike as the curvatures of the reconstructed spike are obtained through fitting second- and higher-order polynomials.

### Spatial compression

It is a known fact in electrophysiology that there are inevitable redundancies in the information acquired through multi-channel, intra-cortical, extra-cellular neural recording from a small volume of the brain. Neural signals recorded in a small volume of the brain share the same LFP in common. Therefore, the LFP recorded on one channel might convey useful information (depending on the application), but it is apparently redundant on all other channels. Moreover, given the rather small distance between neighboring electrodes on a highly-dense microelectrode array, the firing of a given neuron is usually captured on multiple adjacent recording sites (Lopez et al. 2016, 2017; Jun et al. 2017; Herbawi et al. 2017). Spatial compression of multi-channel recordings usually aims at enhancing the data compaction efficiency through reducing such redundancies. The idea of using the whitening transform for reducing redundant activities on neighboring neural channels is first introduced in 2006 (Oweiss 2006). The computationally heavy calculation of signal correlations (requiring multiplications), however, makes this idea impractical at the time. (Yazdani et al. 2014) propose a hardware-efficient implementation for the whitening transform, using which spatial neural signal compression is made possible. In this work, spatial compression is achieved through aggregating redundant spiking activities as well as correlated background noise on one channel (named as the *principal channel*) and removing them from all other channels (referred to as *subordinate channels*). Moreover, the *Multichannel-Baseline-Extraction Decomposition* (*MBED*) technique in (Khazaei et al. 2020) and (Keramatzadeh et al. 2020) suggests that the common baseline (*i.e*., the LFP) is extracted from multiple adjacent channels. The amplitude of baseline variations is usually much larger than that of neuronal activities. Streaming of the extracted common baseline along with channel-specific neuronal activities off the implant results in both loss-less compression and saving significant amount of bit rate by not reporting redundant baseline data multiple times.

It is worth noting that, if applicable, spatial and temporal compressions can be performed on the same multi-channel neural recording implant. In this case, the total compression rate will be the product of the temporal and spatial compression rates (Yazdani et al. 2014). In addition, another technique proposed in (Park et al. 2018) suggests a spatio-temporal technique based on frequency band separation to enable the independent compression LFPs and isolated spikes, aiding in data compression.

A comparative specification summary for the of the existing data compression techniques is presented in Table 1. This summary provides insights into compression efficiency from both signal processing and hardware implementation perspectives.

**Table 1:** Comparative performance summary of the existing data compression techniques

		K.G. Oweiss et al 2007	M.A. Shaeri et al 2011; M.A. Shaeri and A.M. Sodagar 2015	H. Hosseini-Nejad et al 2011	N. Yazdani et al 2014; N. Yazdani et al 2018	S. Farsiani and A.M. Sodagar 2020; S. Farsiani and A.M. Sodagar 2023	M. Nekoui and A.M. Sodagar 2022	Y. He et al 2024	Weltin-Wu and Tsividis Sept. 2013)
**Compression** **method**		Sym. 4	Haar	WHT	DCT	Whitening Xform	Tchebycheff Xform	Salient Sample Extraction	Event-based Spat. Grouping
**No. of Channels**		32	64	128	128	16	256	128	128
**Comp. Measures**	_**CR**_^**a**^	64	116.4	63	69	122	154	272	20
	_**TCR**_^**b**^	N/A	903	504	552	N/A	1232	2174	N/A
**CMOS Process (nm)**		500	130	180	65	180	180	130	65
**Supply Voltage (V)**		N/A	1.2	1.8	1.2	1.8	N/A	1	1.2
**Area/Ch. (mm**^**2**^**)**		0.18	0.0032	0.0128	0.018	0.0093	0.011	0.0028	0.00007
**Power/Ch. (µW)**		94	1.47	0.63	0.74	7.43	N/A	0.164	0.02

^a^Compression Rate

^b^True Compression Rate

## Spike sorting

**Fig. 8:**
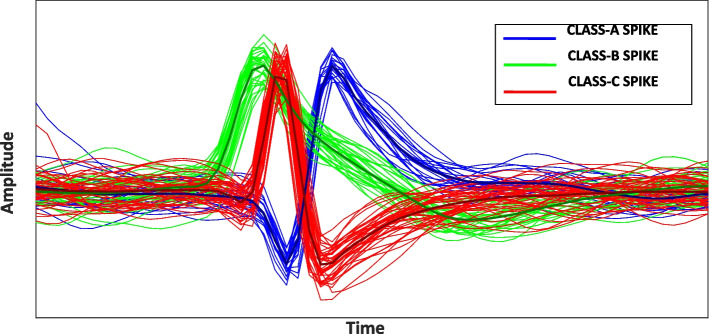
Three spike classes, plotted in green (32 spikes), red (41 spikes), and blue (22 spikes). Dark markers show the representative spike waveform for each class obtained through averaging

It is a known neuroscientific fact that the extra-cellular action potentials recorded from a given neuron are all of the same general pattern in their amplitude variations. From a signal’s perspective, however, when recorded extracellularly, such spikes are not identical as they are contaminated with random noise, as discussed before. Although extracellular spikes recorded from different neurons are meaningfully distinguishable from both the LFP and background noise, their waveforms vary from one neuron to another. Therefore, in an intra-cortically-recorded neural signal, it is assumed that the neural spikes being of a certain waveshape are associated with the firings of a specific neuron. Figure 8 presents multiple occurrences of the neural spikes extracted from a neural signal acquired through *in-vivo,* intra-cortical recording (Quiroga 2020). Disregarding the small random variations caused by random noise, the spikes are of three different general waveshapes, clustered in three *classes* each designated using a specific color.

**Fig. 9:**
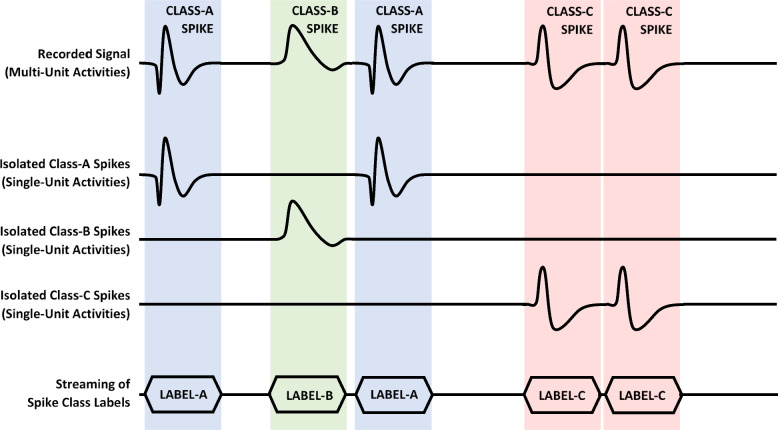
Illustration of the basic concept of spike sorting

In applications such as neuroscience research and brain mapping, acquiring knowledge about the origin of recorded activities is sometimes a necessity. *Spike sorting* is the act of recognizing and then clustering recorded spikes according to the neuron-specific pattern that exists in their waveshapes. After an initial training, upon receiving a detected spike, a spike sorter identifies the *unit* (firing neuron) that has issued that spike and labels the spike with the spike class it belongs to. Figure 9 illustrates the concept of spike sorting, in which the recorded signal captures neuronal activities of multiple units (in this case three units: A, B, and C). Spike sorting discriminates and then isolates spikes into separate *single-unit activities* according to their waveshapes and tags them with class-specific labels. Streaming the ‘label’ of the associated spike class takes significantly less data bits than sending all samples of that spike off the implant. This is why on-implant spike sorting is a much more effective approach to reduce the amount of data telemetered from a high-density neural recording device to the outside world.

A spike sorter operates in two phases: In the *training phase,* the spike sorter is presented with a sufficiently large number of spikes, enabling it to learn and recognize the different spike classes within the data. In the *sorting phase*, the on-implant trained spike sorter receives emerging spikes, isolates them according to the classes they belong to, and tags them with the associated class labels. Considering the limited resources on an implant, especially in terms of hardware and electric power, the training phase is preferred to be realized on the external side of the system (ref. Figure 2a) where hardware resources are virtually unlimited. While on-implant spike sorting must be performed in real time, the training of the spike sorter can be done offline (Yang et al. 2017; Shaeri and Sodagar 2020; Kalantari et al. 2022).

Following spike detection and temporal alignment, discriminative features of the spikes are extracted, using which the sorting of the spikes is performed. There are software tools for spike sorting that implement different algorithms for automatic spike sorting. As an example, the spike sorter introduced in (Pachitariu et al. 2024) offers fully automatic spike sorting, utilizing template matching, drift correction, and GPU-accelerated clustering algorithms, all based on principal component analysis (PCA). While such tools significantly reduce manual intervention and improve sorting accuracy, they may not be practical choices for hardware implementation due to their computational demands.

Analog techniques for sorting and classifying neural data have attracted considerable interest from researchers. For instance, (Hao et al. 2021) proposes ADC-free analog front ends, and leverages Continuous-Time First and Second Derivative Extrema (CT-FSDE) for feature extraction. These approaches aim to achieve enhanced classification accuracy through quantizer-free and clockless operation. However, it is important to note that analog implementations may result in higher costs in terms of silicon area (and sometimes in power consumption) compared to digital implementations, which can be a limiting factor. Expanding on the perspective of on-chip implementations of spike sorting algorithms, both supervised (Do et al. 2019; Zeinolabedin et al. 2022), and unsupervised (Zamani et al. 2018; Valencia and Alimohammad 2019; Karkare et al. 2013; Chen et al. 2023) approaches have been developed to meet the requirements of real-time applications. These implementations not only enhance the efficiency of neural recording systems in terms of real-time application demands, including low-latency signal processing, reliable spike detection, and effective neural data handling for dense neural interfaces, but also offer benefits such as reduced computational complexity, lower power consumption, and improved scalability.

Online sorting algorithms based on the L1-norm distance (Karkare et al. 2013), geometry-aware OSort (Geo-OSort) (Chen et al. 2023), hierarchical adaptive means (a modified version of the K-means algorithm) (Paraskevopoulou et al. 2014), oblique decision tree (Yang et al. 2017), salient feature selection and window discrimination (Shaeri and Sodagar 2020), and correlation-coefficient-based spike sorting (Kalantari et al. 2022) are examples of hardware-efficient, online, on-implant spike sorting algorithms reported in the literature. Moreover, (Seong et al. 2021) presents an online spike sorting processor that employs an L2-normalized convolutional autoencoder for feature extraction, combined with a similarity-based K-means clustering algorithm. This algorithm conditionally updates cluster centroids based on cosine similarity, ensuring enhanced convergence and improved classification accuracy. Additionally, (Valencia and Alimohammad 2019) reports an efficient hardware architecture for spike sorting using online template matching, achieving substantial power and area reductions through software/hardware co-design and template optimization while maintaining high sorting accuracy.

It is true that sorted spikes (single-unit activity) measure neural activity at the level of individual neurons, which is highly valuable for neuroscience studies. However, it is worth noting that in BCI applications, some researchers believe that spike sorting can be bypassed, with threshold-crossing rate (a.k.a., multi-unit activity) being a suitable alternative for analyzing neural population dynamics (Trautmann et al. 2019). In these studies, spike counts are typically measured in 10–50 ms time bins and are often smoothed using a Gaussian filter to represent neural activity for decoding purposes (Shaeri et al. 2024). Alternatively, spiking band power (SBP) or the entire spiking activity (ESA), which captures low-frequency neural signals, may also be beneficial in these applications (An et al. 2022; Ahmadi et al. 2021).

## On-Implant signal processing: hardware-implemented vs. hardware-embedded

It was previously mentioned that signal compression can be performed almost anywhere in the signal path on a neural recording implant, as illustrated in Fig. 2b. From a system-level perspective, two major approaches are taken to compress neural signals on brain implants: *hardware-implemented* signal compression, and *hardware-embedded* signal compression. The former is the class of signal compression techniques, for the realization of which a special-purpose processor is designed. The latter refers to the realization of signal compression in a circuit block that is primarily in charge of a different task in the system. All the processing techniques discussed so far are categorized under the hardware-implemented class.

### Compression on recording front-end

**Fig. 10:**
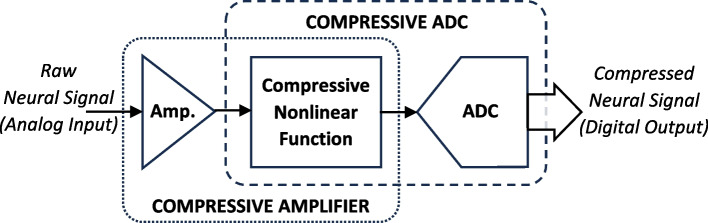
Realizing a compressive recording front-end by adding a compressive nonlinear function along the analog signal path as reported in (Judy et al. 2010, 2014)

Examples of hardware-embedded signal compression techniques that have been implemented on the recording front-end block can be explained as follows: In the neural recording front-end, as illustrated in Fig. 10, adding a proper nonlinear function can help realize neural signal compression. If combined with the pre-amplifier, the result will be a nonlinear amplifier. The logarithmic amplifier reported in (Chelbi and Sawan 2013) is an example, which effectively compresses the signal in the analog domain during amplification. If grouped with the analog-to-digital converter, a compressive analog-to-digital converter (ADC) will be resulted. The nonlinear ADC in (Judy et al. 2010, 2014) has an anti-logarithmic transfer characteristic that maps the input analog amplitude to a compressed digital code at the output. With a physical resolution of 8 bits at the output, this ADC digitizes the background noise with a resolution of 3 bits and large neural spikes with a resolution of up to 10.6 bits. The fact that action potentials (spikes) are sparse events in a neural signal makes this ADC significantly data and power efficient.

**Fig. 11:**
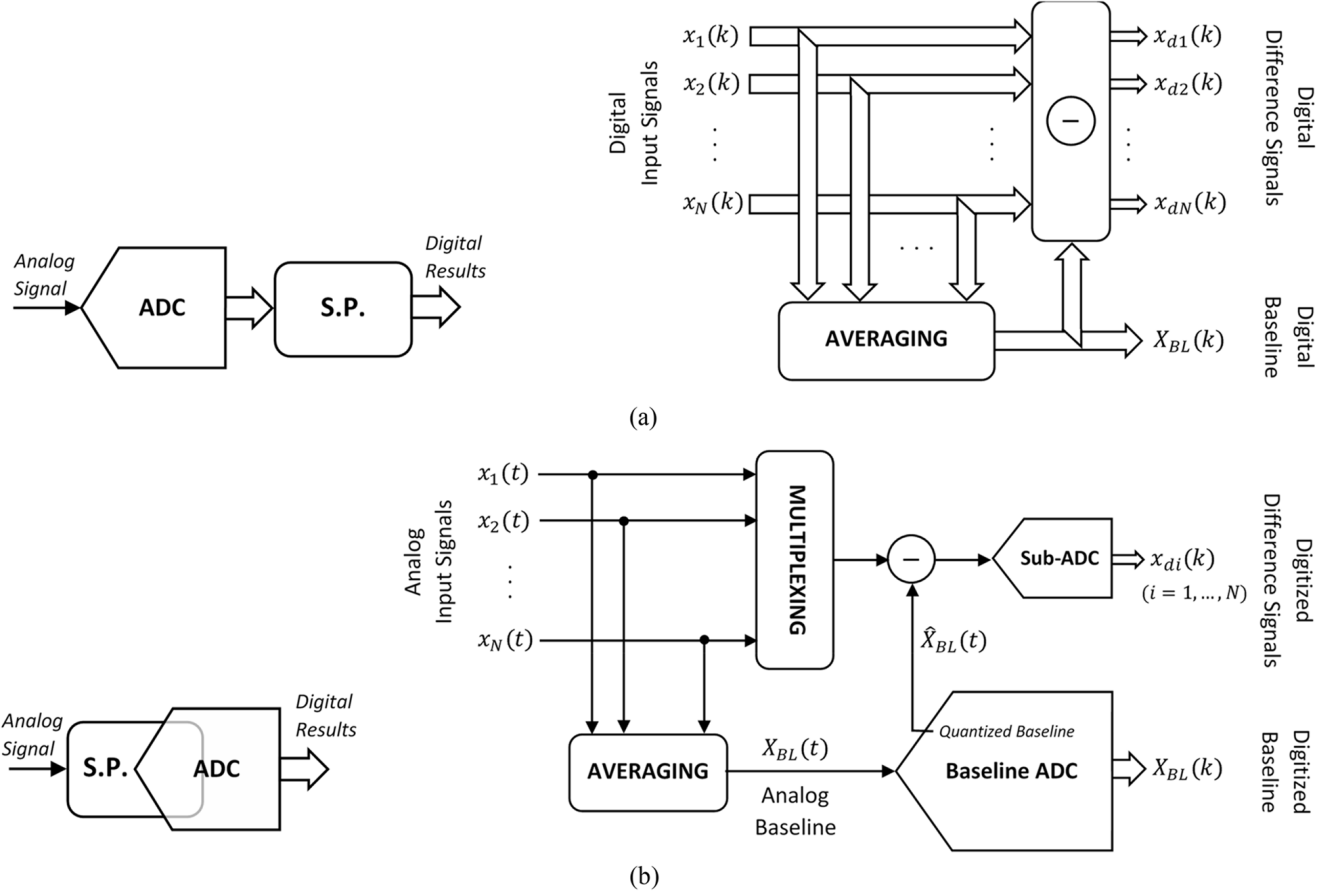
The MBED technique: (**a**) Hardware-implemented realization (Khazaei et al. 2020) & (Keramatzadeh et al. 2020), (**b**) Hardware-embedded realization (Khazaei et al. 2019)

An example that highlights the difference between hardware-implemented and hardware-embedded signal processing is the two realizations reported for the MBED spatial compression technique. Figure 11a pictures the hardware-implemented realization of this technique. A stand-alone signal processing block (S.P.) follows the digitization process and performs the MBED technique on multiple channels of concurrent neural signals in the digital domain 2014 Khazaei, et al. 2020; Keramatzadeh et al. 2020). As reported in (Khazaei, et al. 2019) and demonstrated in Fig. 11b, this same signal processing technique is employed to design a multi-channel ADC. The common LFP component is first acquired and then separated from the input neural signals in the analog domain, and then digitized using the Baseline ADC. A quantized version of the baseline (in analog form) is subtracted from the neural signals, leaving behind LFP-free ‘difference signals’, which are much smaller than the input signals in amplitude range. This significantly reduces the real estate (in terms of silicon area and power consumption) required for the digitization task.

As a different instance of hardware-embedded spatial data compression, the Wired-OR ADC structure, with competition among recording sites, presents lossy compression capabilities, effectively achieving data compression in multi-channel recording systems (Yan, et al. 2023). Additionally, the literature suggests non-traditional ADC techniques such as event-driven level crossing ADCs (Assche and Gielen 2020) , adaptive sampling ADCs (Chen et al. 2022), and adaptive resolution ADCs (Wu and Tsividis 2013), all facilitating a reduction in the output data.

### Compression on wireless interfacing back-end

Further compression of recorded signals can also be embedded in the wireless transmission back-end hardware. For instance, it has been shown in (Shaeri et al. 2011; Shaeri and Sodagar 2015; He et al. 2024); and (Farsiani and Sodagar 2022) that the data framing protocol can be arranged in such a way that the outgoing data is exempt from additional data usually envisioned for synchronization and/or addressing purposes.

**Fig. 12:**
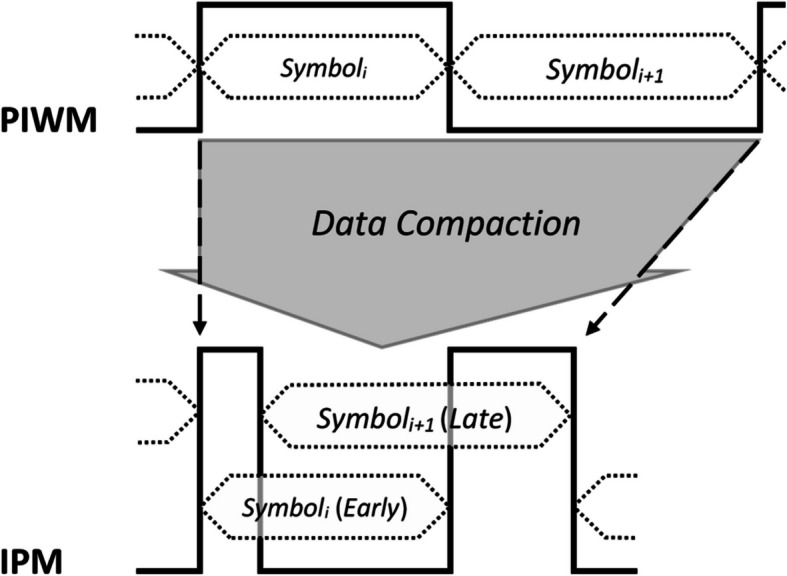
Compressive telemetry through compact baseband modulation (encoding) of data using the Intertwined Pulse Modulation (IPM) technique (Farsiani and Sodagar 2022)

As a totally different attempt at the wireless transmission step, an innovative anisochronous baseband modulation scheme has been proposed in (Farsiani and Sodagar 2022) that takes advantage of the temporal properties of neural signals and transmits the recorded data in a more compact way. Referred to as *Intertwined Pulse Modulation* (*IPM*), this scheme maximally overlaps every two consecutive symbols (named as ‘early’ and ‘late’ symbols), through which data compaction is made possible. Figure 12 compares the IPM method with Pulse Interval and Width Modulation (PIWM), which itself is a highly symbol-rate efficient pulse-based anisochronous baseband modulation scheme. In IMP, symbol overlaps, and consequently, data compaction is maximized when the early and late symbols are close in value. Interestingly, this condition holds true both for background noise and throughout the spike time course.

## Reduction of circuit/computational complexity

In on-implant signal processing, there are techniques that can be used for circuit complexity reduction with no or insignificant impact on the accuracy of the processing results.

### Simplification of calculations

A preliminary step towards reducing circuit and computational complexity is the simplification of calculations with no penalty on the accuracy of the achieved processing results. As mentioned before, the traditional approach to automatic spike detection is comparing the neural spike with an integer multiple of the standard deviation of the background noise. Since the signal is already squared for the calculation of standard deviation, it is suggested in (Barati and Sodagar 2010) that spikes are detected by comparing the squared signal with the variance of the background noise. This eliminates the need for square rooting the variance of the background noise and results in reducing both computational and complexities with no effect on the accuracy of spike detection. Alternatively, based on the assumption that the background noise follows a Gaussian distribution, the standard deviation can be estimated from the number of samples exceeding it, enabling calculation without the need for multipliers (Guo et al. 2022).

In transform-based spike compression methods such as (Shaeri and Sodagar 2015) and (Hosseini-Nejad et al. 2011), multiplication of the transform matrix by a constant factor is eliminated from taking the transform on the implant side. This can be compensated for when taking the inverse transform of the signal on the external side, if needed. This simplifies the calculations on the implant side with no costs on the overall signal-level performance of spike compression.

The spike sorting approach reported in (Kalantari et al. 2022) works based on comparing the resemblance of each emerging spike to the representatives of all spike classes. This is done through calculating the associated correlation coefficients, sorting them in a descending order, and finding the largest coefficient. Formula-wise, all the coefficients have the standard deviation of the samples of the spike under study in their denominator. This common term, apparently, does not contribute to the result of the aforementioned sorting and comparison, and can therefore be removed. This significantly simplifies the on-implant spike sorting process in the sorting phase.

### Approximation in calculations

In neural signal processing, there are several reasons why we can tolerate some degree of inaccuracy in our computations. For instance, *within-class variability* of the spikes recorded from the same neuron is caused by the random background noise. This noise deviates the waveshapes of same-class spikes from the noiseless representative spike of that class. In the same way we accept this deviation, some extent of computational error is acceptable in spike processing with no considerable effect on the integrity or quality of the signal. (Yazdani et al. 2018) Yazdani et al., reduces the computationally-heavy calculation of the correlation between two signals (requiring multiplications) to finding an approximate form of correlation (implemented merely using addition and shift operations).

Another example of approximate computing in this context is the *Compact Agile Tchebycheff* (*CATCH*) *transform* proposed in (Farsiani and Sodagar 2023). This is an approximate form of the original Tchebycheff transform that is proposed for the simpler hardware implementation it offers. The hardware efficiency in this work comes at the price of a tolerable extent of energy leakage among the transform coefficients that are of least significance in carrying the signal energy.

### Data truncation

During digital signal processing, the length of data words sometimes unnecessarily increases. For instance, when processing signals with N bits of resolution, if the signal is squared or if it is multiplied by another signal, the length of the result will be 2 N bits. In this case, some of the least-significant bits (LSBs) might be ignored with negligible effect on the accuracy of computations. Some other times, the range of the data is significantly less than the capacity of the number of bits envisioned for it. As a result, some of the most-significant bits (MSBs) might remain unused and can, therefore, be ignored without affecting the processing results. To conclude, there are situations where the length of data words can be reduced by discarding some of the data bits (either unused bits or those of insignificant information) (Shaeri and Sodagar 2015; Yazdani et al. 2014). Referred to as *truncation* (or *quantization*), this action can result in considerable data reduction, hardware simplification, or both (Shaeri and Sodagar 2015).

Truncation at a higher level is employed in (Farsiani and Sodagar 2023), where some of the CATCH transform coefficients are discarded. In its original form, this transform maps a window of 8 signal samples in the time domain to 8 coefficients in the transform domain. Using a well-developed design procedure, the CATCH transform concentrates the majority of the signal energy in the first few coefficients. As a result, it is shown that even after truncating the last 6 coefficients, neural spikes are reconstructed in the time domain with negligible loss of signal energy.

### Proper data format

Compared to floating-point calculations, *fixed-point arithmetic* is often preferred for on-implant signal processing as it significantly reduces hardware complexity and power consumption. While offering less precision and dynamic range, the fixed-point style is still capable of providing sufficient accuracy for a wide variety of biological signal processing tasks (Menard et al. 2006).

Another method often preferred in on-implant systems is *bitfields* or *compact binary formats*, which store multiple logical or categorical data points within a single word, significantly reducing memory usage and simplifying operations. This approach, unlike the traditional data formats that allocate a full word for each variable, offers benefits such as lower memory consumption, faster data access, and reduced power requirements. This makes the proposed format highly efficient for resource-constrained systems. While accessing individual bits can introduce some extent of complexity and portability concerns across platforms, the overall reduction in memory and power consumption still makes this approach advantageous (Patel and Bleakley 2016).

### Artistic design recipes

When designing a circuit for the realization of a given processing task, there are occasions where an experienced circuit designer composes a recipe of concepts at both signal and circuit levels to come up with a hardware-efficient design. For instance, in digital signal processing, there are situations where a signal should be multiplied by a constant factor. Because of its high computation and hardware costs, a digital multiplier is not a proper choice, especially if there is a number of such multiplications in the processing task at hand. In this case, instead of using a multiplier, one can scale the signal by a proper combination of right/left shifts, additions, and subtractions. For instance, consider a constant factor of 5.875. Using the *canonic signed digit* (*CSD*) *representation* (Parhami 2010), this factor can be written as: $4+2-1/8$. Therefore, multiplying a signal by this factor can be easily implemented by shifting the signal to the left by 1 and 2 bits, shifting it to the right by 3 bits, and adding/subtracting the results. Hardware implementation of this scaling is even further simplified if the right/left shift operations are done through ‘shifted bit-wise wirings’ rather than employing traditional shift registers. The predictive ADC presented in (Namavar et al. 2022) realizes the coefficients of a predictive filter using this technique.

## Quantitative measures

In on-implant signal processing, the merit of processing tasks and operations is not only in the processing itself, it is also determined in how well they are implemented in hardware. As such, the success and merit of such tasks is assessed using both *signal processing measures* and *hardware measures.* Moreover, given the nature of the signals being processed, variabilities such as spike classes and random noise contamination need to be taken into consideration when assessing the performance of signal processing on brain-implantable devices.

### Signal quality

In intra-cortical neural recording, the quality of a recorded signal is usually expressed in terms of the strength of neural spikes in comparison with the background noise. Quantified using the *signal-to-noise ratio* (*SNR*), there are different definitions for this figure in neural recording. The common definition for the SNR is the ratio of signal power to the noise power (Rieke et al. 1997). In (Khodagholy et al. 2013), the SNR is introduced as the ratio of the spike peak-to-peak amplitude to the root-mean-square (RMS) value of the background noise. With this definition, a good neural signal has a typical SNR of 5 ~ 10 or even higher. The SNR is sometimes defined as the ratio of the spike RMS value to the RMS of the background noise (Kuzum et al. 2014; Blaschke et al. 2017).

### Signal processing measures

Depending on the category of signals processing (discussed in previous sections), success and quality of the processing is assessed using a variety of metrics (Bishop 2006).

### Metrics for spike recognition

**Fig. 13:**
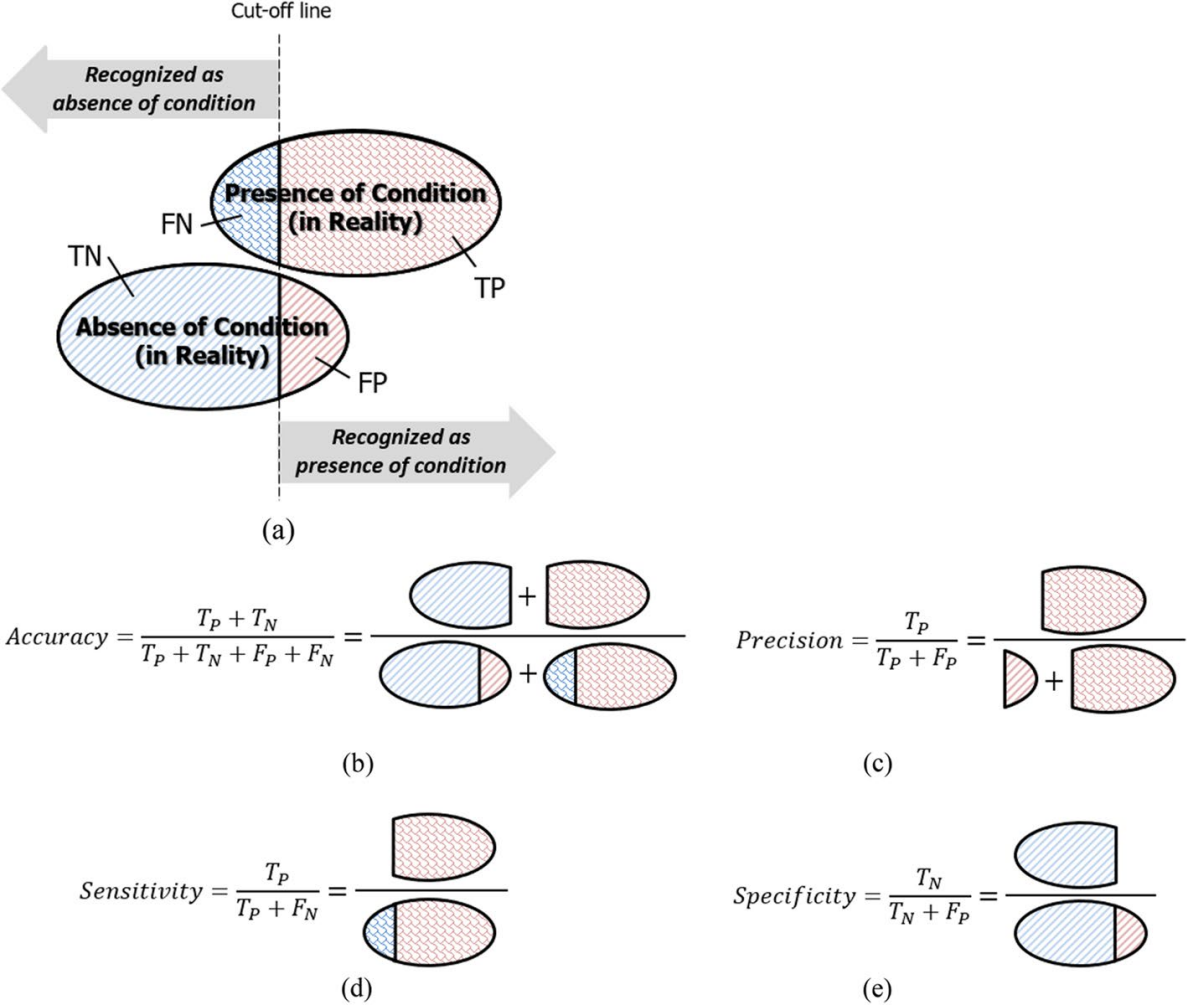
Metrics for spike recognition (*i.e*., detection or sorting): (**a**) four measures to quantify the success or failure of spike recognition, (**b**) accuracy: an overall measure of how well the task performs, (**c**) precision: a measure of the quality of positive recognition, (**d**) sensitivity: a measure of the quality of the recognition of actual spikes, and (**e**) specificity: a measure of the quality of the recognition of non-spikes or the correct identification of spikes that do not belong to the spike class

Spike detection and spike sorting are similar processing tasks in that they both aim to identify and recognize spikes. Spike detection recognizes the presence of spikes, and spike sorting recognizes the type of spikes. The former discriminates spikes from the background noise and the latter isolates spikes according to their waveshapes. In this context, as illustrated in Fig. 13a, the success or failure of recognition in the processing task at hand (*i.e*., detection or sorting) is expressed using one of the following four measures:

#### True Positive (TP)

The number of instances where the task correctly identifies the presence of a spike (in spike detection) or correctly associates a spike with the appropriate class (in spike sorting).

#### True Negative (TN)

The number of instances where the task correctly identifies the absence of a spike (in spike detection) or correctly determines that a spike does not belong to a particular class (in spike sorting).

#### False Positive (FP)

The number of instances where the task incorrectly identifies the presence of a spike (in spike detection) or incorrectly assigns a spike to a class it does not belong to (in spike sorting).

#### False Negative (FN)

The number of instances where the task incorrectly identifies the absence of a spike (in spike detection) or fails to assign a spike to its correct class (in spike sorting).

Using the above four measures, the four metrics shown in Fig. 13b-e are introduced with the following definitions to quantify the performance of the task at hand:

##### Accuracy

The proportion of correctly identified instances (true positives + true negatives) out of all instances.

##### Precision

The proportion of correctly identified positive instances (true positives) out of all instances that are identified as positive instances (true positives + false positives).

##### Sensitivity

The proportion of correctly identified positive instances (true positives) out of all actual positive instances (true positives + false negatives).

##### Specificity

The proportion of correctly identified negative instances (true negatives) out of all actual negative instances (true negatives + false positives).

##### F1-Score

The harmonic mean of Precision and Sensitivity.

##### ROC Curve

The plot of true positive rate vs. false positive rate, known as the Receiver Operating Characteristic (ROC) curve, and its area under the curve.

##### Adjusted Rand Index (ARI)

A metric used in unsupervised spike sorting that evaluates the similarity between clustering results and ground truth by counting matching spike pairs and adjusting for random agreement.

##### Normalized Mutual Information (NMI)

An information-theoretic metric used in unsupervised spike sorting which quantifies the mutual dependence between predicted clusters and ground truth labels.

In the literature, spike detection accuracy and spike sorting accuracy are much more commonly used than the other three measures (Yang et al. 2017)-(Chen et al. 2023).

*Metrics for Compression*-In neural signal compression, the extent of reduction in the data as a result of applying a compression technique is usually measured by *compression rate* (*CR*). The CR is defined as the ratio of the number of bits in the raw data to the number of bits after compression:

1 $$CR=\frac{\#\ of\ Bits\ in\ the\ Raw\ Signal}{\#\ of\ Bits\ in\ the\ Compressed\ Signal}$$

As mentioned before, in a wide spectrum of neural signal compression techniques, a great deal of data compression is achieved through spike extraction, and a portion of it comes from data reduction techniques such as spike compression, truncation, and compact data representation. The portion of the compression rate that is achieved through spike extraction proportionally depends on the *firing rate* of spikes in the neural signal being compressed. This evidently makes the CR a function of both the signal compression technique used and the firing rate of spikes. To be independent from the spike firing rate, the *true compression rate* (*TCR*) is introduced in (Shaeri and Sodagar 2015) as:

2 $$TCR=CR\times \frac{Spike\ Firing\ Rate}{1\frac{Spike}{Sec.}}$$

This is a measure that can be taken as either the CR normalized to the spike firing rate of the signal, or the CR for a firing rate of 1 spike per second.

### Metrics for reconstruction error

Let us assume that a recorded (original) spike, *y*(*t*), is sampled and digitized before it is processed (*e.g.*, compressed) on the implant side of a neural recording system. The result is a discrete-time digital signal, *y*(*k*). Suppose that we take a *K*-sample slot of the signal, send it off the implant through wireless transmission, and reconstruct the signal on the external side, $\widehat{y}\left(k\right)$. The *reconstruction error*, ${e}_{RMS}$, is a measure quantifying the change in the signal as it passes through the ‘processing-retrieval’ (*e.g.*, compression-decompression) process. Traditionally defined as the *Euclidean distance* between the reconstructed signal and the original (recorded) signal (Antoniou 2016), the reconstruction error is calculated as:

3 $$e_{RMS}=\sqrt{\frac1K{{\;{\sum\nolimits_{k=1}^K}}}\left[ y\left(k\right)-\widehat y\left(k\right)\right]^2.}$$

The reconstruction error is sometimes normalized to the peak-to-peak amplitude of spikes existing in the neural signal in order to give a sense of signal change independently from the absolute signal amplitude (Caro-Martín et al. 2018).

According to (Oppenheim 2009), the average absolute difference between corresponding samples in the original and reconstructed signals is introduced as the *Mean Absolute Error* (*MAE*):

4 $$e_{MEA}=\frac1k{\textstyle\sum_{k=1}^K}\left|y\left(k\right)-\widehat y\left(k\right)\right|.$$

### Hardware measures

Evaluating the hardware in signal processing for implantable brain devices requires standardized metrics to compare various designs. This section discusses methodologies for power consumption and silicon area evaluation and highlights the importance of normalization techniques to account for variations in CMOS technology, supply voltage, and channel configurations, ensuring fair comparisons of power consumption and silicon area across different approaches.

Given the limited electric power available to a brain implant, *power consumption* is one of the most critical metrics for the assessment of the suitability of a signal processing hardware for on-implant implementation. *Silicon area consumption* is another metric that determines both the hardware complexity and the physical size of the hardware implementation of the on-implant signal processor. In some works, alongside the silicon area, circuit complexity is expressed in terms of the number of multiply-accumulate units (MACs), or the number of gates or transistors used (Paraskevopoulou et al. 2014; Guo et al. 2024). In the context of high-density neural recording implants, the number of channels varies from work to work. Therefore, both power and area of on-implant signal processors are usually normalized to the channel count, and power-per-channel and area-per-channel metrics are reported for fair comparisons 2024;(Hao et al. 2021; Do et al. 2019; Zeinolabedin et al. 2022).

It should be added that for even more reasonable comparison, the work in (Shaeri and Sodagar 2015) moves one more step forward and projects the power-per-channel and area-per-channel values to the same microfabrication process.

## Challenges and future directions

Development of miniaturized complex tools such as brain implants is a highly challenging multi-dimensional task, mutually handled by specialists in engineering and biology. As progress is declared on the tools side, new hopes and expectations for higher levels of functionality and performance are expressed on the application side, which serve as a strong drive force towards more progress. However, any enhancement in the function or performance of such devices adds to the existing ‘technical’ and ‘technological’ challenges faced.

### Challenges

Handling the huge amount of data recorded by cutting-edge, high-density arrays is and will remain a serious challenge. With the enhancement of the recording capacity of brain implants, higher power demand by implants on one hand and restrictions in supplying the required power to implants on the other hand will direct designers towards circuit-level techniques such as extremely low-power design, system-level methods such as resource sharing and power saving and management, and highly efficient processing approaches at the signal level.

For extremely high-density implants, the limitation of wireless transmission bandwidth becomes a bottleneck. Transmitting heavy traffic of recorded data off the implant will, therefore, no longer be a viable option. As a result, brain implants will need to handle even more complex processing tasks, with only the high-level results being telemetered to the outside. While this will undoubtedly introduce additional challenges, particularly in terms of power consumption, it appears to be an inevitable and difficult decision. The cost of this choice will be offset by greater efforts in circuit design.

### Future directions

To address the challenges discussed above and work towards more capable implants, the following directions will be among the key focal areas for research and development in on-implant signal processing:

#### Hardware/processing co-design

As extensively discussed in this paper, signal processing and hardware implementation are equally essential to achieving ‘implant-grade’ merit for on-implant signal processing. This becomes more critical as the recording capacity of brain implants increases. Therefore, the concept of hardware/processing co-design is expected to be able to respond to the inevitable need for achieving a higher level of signal processing efficacy through mutual optimization of both signal processing and hardware implementation.

#### Smart signal processing

The recent flourishing of artificial intelligence (AI) is anticipated to open new horizons in computation and potentially introduce novel paradigms that help enhance the efficacy of neural signal processing in brain implants. Smart processing of neural signals could leverage AI approaches and techniques to further enhance both processing and hardware performance, or to improve the efficacy of the hardware/processing co-design concept discussed earlier.

#### Protected operation

Brain-implantable devices are expected to become an integral part of our lives in the near future, initially for medical and prosthetic applications and potentially for cognitive enhancement and even more advanced purposes. As these devices establish wireless communication with the outside world, several concerns are anticipated to emerge as critical priorities. These include the privacy of brain data, the security of implantees against intrusions and cyber-attacks, and the safety of implantees when exposed to man-made and natural interferences such as MRI scans, X-ray inspections, and solar storms. Addressing these issues will be essential as brain implants transition from research prototypes to commercial products.

#### Personalized signal processing

Despite the commonality of the general aspects of brain activities and neuronal signals, the signals acquired by a neural recording device vary in details from subject to subject and even from one recording session to another. Fitting the processing approach to the person-specific details and patterns existing in the recorded signal can potentially result in higher performance, lower complexity, or both. This can be realized through employing adaptive techniques, optimization methods, or the training of processing algorithms to make them maximally appropriate for the specific processing scenario.

## Conclusion

A brief review of hardware-efficient neural signal processing is presented, which is focused on high-density, brain-implantable, neural recording microsystems. In addition to their effectiveness in signal processing, fully-integrated hardware implementation, low power consumption, and real-time operation are among the key requirements that define the suitability of such techniques for on-implant neural signal processing.

## Data Availability

No datasets were generated or analysed during the current study.

## Footnotes

1 Please note the distinction between data, signal, and information throughout this paper: Data refers to raw numbers, measurements, or observations; a signal is a sequence of data samples in time, frequency, space, or other domains; and information is the meaningful interpretation derived from processing and analyzing the data
